# When the Benign Pneumatosis Intestinalis Becomes No Longer Benign: A Rare Case of Bowel Perforation in a Patient with Systemic Sclerosis

**DOI:** 10.1155/2018/5124145

**Published:** 2018-08-26

**Authors:** Jian Guan, Alvina Munaf, Alric V. Simmonds, Irteza Inayat

**Affiliations:** ^1^Department of Internal Medicine, Graduate Medical Education, Florida Hospital, 2501 N. Orange Suite 235, Orlando, FL 32804, USA; ^2^Department of General Surgery, Graduate Medical Education, Florida Hospital, 2415 N. Orange Ave., Suite 400, Orlando, FL 32804, USA; ^3^Central Florida Hepatology & Gastroenterology, Florida Hospital, 2415 N. Orange Ave., Suite 200, Orlando, FL 32804, USA

## Abstract

Systemic sclerosis is a multisystem disease featured with autoimmunity and organ fibrosis. Although gastrointestinal (GI) tract involvement is common in patients with systemic sclerosis, colonic perforation is extremely rare. Benign pneumatosis intestinalis, a phenomenon more frequently seen in rheumatologic conditions, makes the diagnosis of colonic perforation even more challenging. We report a unique case of colonic perforation in a patient with chronic systemic sclerosis. This patient initially presented with mild abdominal pain and hematemesis. Urgent upper endoscopy was unremarkable and radiology showed stable pneumatosis intestinalis. Due to worsening abdominal pain, laparotomy exploration was performed and colonic perforation with transmural ischemic necrosis was found.

## 1. Introduction

Systemic scleroderma or systemic sclerosis is a chronic multisystem disease featured with generalized vasculopathy, activation of immune response, and progressive fibrosis of the skin and internal organs [1]. Gastrointestinal (GI) tract involvement is very common in patients with systemic sclerosis and dysmotility is the primary manifestation [2]. GI Symptoms are usually nonspecific, including dysphagia, heartburn, nausea, vomiting, abdominal pain, and distention [3]. It is not uncommon to see chronic bowel dilation, pseudo-obstruction, and benign pneumatosis intestinalis on imaging, but these conditions are typically managed conservatively [2]. The bowel perforation is a rare but serious complication of systemic sclerosis with GI involvement [4, 5]. Timely and accurate differentiation of true bowel perforation from benign pneumatosis intestinalis can be challenging.

## 2. Case Description

**Figure 1:**
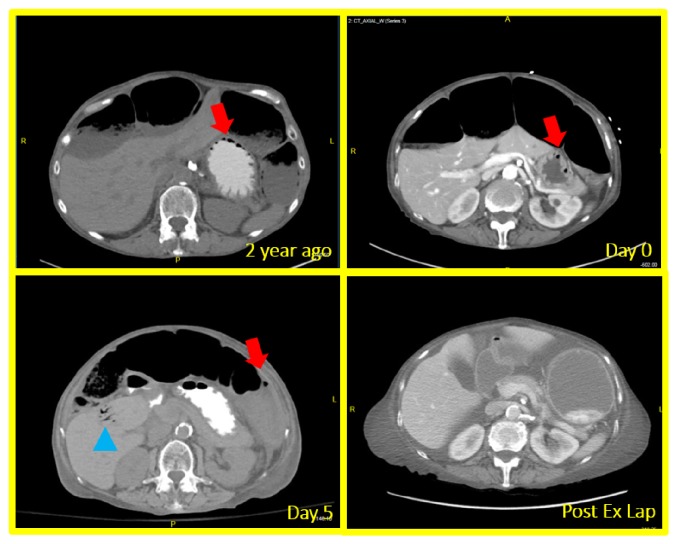
CT of abdomen revealed chronic bowel dilation and pneumatosis intestinalis ((a) and (b)); emergency of portal vein gas and worsening pneumatosis intestinalis (c); improvement after subtotal colectomy (d). Red arrow: pneumatosis intestinalis; blue arrow: portal vein gas.

**Figure 2:**
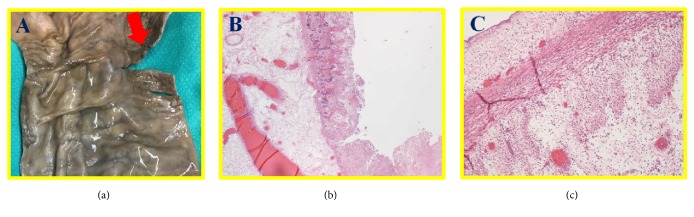
Perforation site in the ascending colon. (a) Gross image of perforation site in ascending colon. (b,c) H&E staining revealed the extensive mucosal necrosis or ulceration and overlying fibrinopurulent exudate.

**Figure 3:**
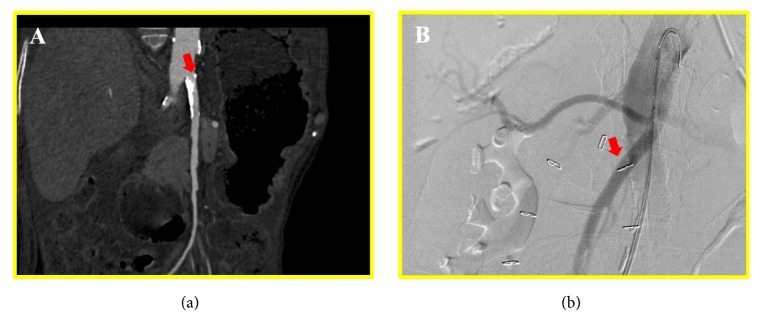
CT angiography abdomen revealed stenosis of mesenteric artery (a). Arteriogram indicated patent mesenteric artery (b). Arrow: mesenteric artery.

A 78-year-old woman with a long-standing history of anti-centromere antibody positive systemic sclerosis with limited skin involvement (CREST syndrome) initially presented to emergency room (ER) for a one-day history of hematemesis and mild abdominal pain. Her medical history was remarkable for peripheral artery disease and duodenal arteriovenous malformation. At presentation, her vital signs were within normal limits and her physical examination revealed mild periumbilical tenderness and decreased bowel sounds without significant signs of peritonitis. Laboratory studies revealed a WBC count of 29X10_ _^∧3^/*μ*l and a hemoglobin of 4.7 g/L which acutely declined compared with her baseline hemoglobin of 9.5 g/L. Renal/liver function tests were within normal limits. Elevated venous lactate level was noted. Patient received intravenous fluid resuscitation and empirical piperacillin/tazobactam as well as 2 units of red blood cells transfusion in ER. A subsequent computed tomography (CT) scan of the abdomen that showed significantly dilated colon and pneumatosis intestinalis (Figure 1). No surgical intervention was deemed necessary per general surgery consultation, considering that the pneumatosis intestinalis was stable compared to the previous CT findings two years ago and the absence of acute peritonitis sign. Patient was then admitted to ICU for close monitoring and management of acute on chronic anemia from GI bleeding. Urgent esophagogastroduodenoscopy (EGD) only identified Barrett's-type esophageal mucosa, erosive gastritis without actively bleeding lesion. Patient was discharged home on the 4th day of hospitalization after tolerating liquid food. However, she was brought back to ER for severe lower abdominal pain just a few hours after discharge. The abdominal pain was sharp, constant, and nonradiating. No melena or hematochezia was reported. On examination, absent bowel sounds and abdominal distention without rebound tenderness or guarding were noted. Her laboratory evaluation was significant for leukocytosis and hypokalemia. Repeated CT scan of abdomen/pelvis showed distended small and large bowel loops, portal venous gas, and pneumatosis within the jejunum and colon (Figure 1). Clinical diagnosis of bowel perforation was made, for which patient underwent emergent exploratory laparotomy. Patchy necrosis throughout the large intestine and a perforated lesion in the ascending colon were identified during the operation. Subtotal colectomy, omentectomy, and end ileostomy were performed. Pathological findings were consistent with colonic perforation with transmural ischemic necrosis and fibrosis (Figure 2). CT angiography and conventional angiogram were performed to determine the culprit artery for bowel ischemia and identified a noncritical stenosis in superior mesenteric artery (Figure 3). Patient was then transferred to ICU for postoperative care. Patient's recovery was complicated with ventilation dependent respiratory failure and another episode of bowel perforation. Given her advanced age, comorbidities, and poor prognosis, end-of-life care was discussed with family members, and they decided to initiate comfort care after a prolonged hospital stay.

## 3. Discussion

Although systemic sclerosis is generally perceived as an orphan disease, its incidence was found to be comparable with the incidences of other well-known diseases such as esophageal cancer [6]. Although exact mechanism of systemic sclerosis remains unclear, [7] evidence suggests that autoimmunity mediated endothelial cell injury, ischemia-reperfusion injury, and generation of reactive oxygen species and proinflammatory cytokines lead to vasculopathy and fibrosis in systemic sclerosis.[7]

GI tract involvement is seen in nearly 90% of patients with systemic sclerosis [3]. Dysmotility in systemic sclerosis is caused by abnormal compliance of bowel wall and reduced contractile function due to myopathy, neuropathy, and fibrosis [2]. Among all of GI complications of systemic sclerosis, pneumatosis intestinalis is probably the most challenging one to diagnose and manage, partially due to the unspecificity of its clinical presentation and potential detrimental consequence [8]. Pneumatosis intestinalis, defined as a cluster of intramural air in small or large intestinal wall, is a relatively infrequent finding and typically associated with secondary cause including autoimmune disease, trauma, and inflammatory diseases [9]. Close monitoring and conservative management such as gastric decompression, bowel rest, and antibiotics are typically recommended.

The bowel perforation is an extremely rare but detrimental complication of systemic sclerosis with GI tract involvement. The common risk factors of bowel perforation including atrophy of intestinal wall, stercoral ulceration from chronic dilation, and fecal impaction are frequently present in patients with systemic sclerosis. The first two cases of bowel perforation in patients with systemic sclerosis were reported in 1970s [4, 5]. More recently, a few reports have linked the use of octreotide or complication of gastrointestinal procedure with bowel perforation [10].

**Figure 4:**
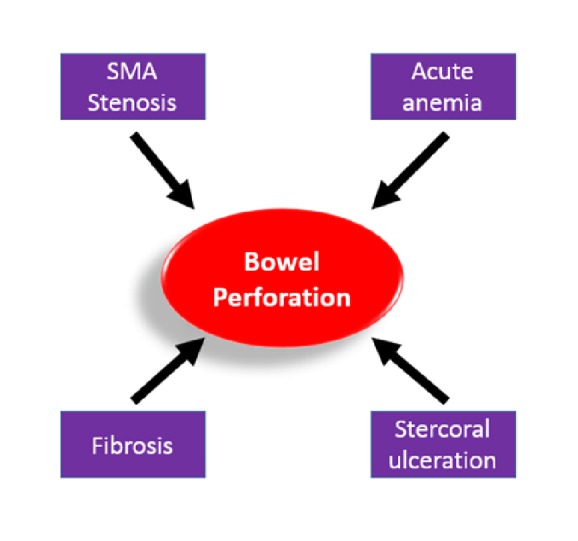
Scheme of the factors contributing to the bowel perforation in this case.

In our case, the cause for the bowel perforation was probably multifactorial (Figure 4). Atrophy of intestinal wall secondary to vasculopathy, inflammation, and fibrosis, as well as chronic dilation induced stercoral ulceration, might be the driving forces for the development of bowel perforation. Acute anemia from GI bleeding in the setting of preexisting atherosclerotic disease involving superior mesenteric artery led to acute bowel ischemia, which exaggerated the process of bowel perforation. There are several learning points in this unique case. Physicians should fully assess the overall clinical manifestation and risk factors, rather than being falsely reassured by radiologically “stable” pneumatosis intestinalis. Premature discharge in her first hospitalization could have been avoided if we had a high suspicion of a ruptured viscus rather than a benign pneumatosis intestinalis.

To sum up, benign pneumatosis intestinalis can be asymptomatic and managed medically in patients with systemic sclerosis; however, bowel perforation needs to be considered in the differential diagnosis in selected cases.

## Data Availability

The data used to support the findings of this study are available from the corresponding author upon request.
